# Review of the Clinical Evidences of Modulated Electro-Hyperthermia (mEHT) Method: An Update for the Practicing Oncologist

**DOI:** 10.3389/fonc.2019.01012

**Published:** 2019-11-01

**Authors:** Attila M. Szasz, Carrie Anne Minnaar, Gyongyver Szentmártoni, Gyula P. Szigeti, Magdolna Dank

**Affiliations:** ^1^Cancer Center, Semmelweis University, Budapest, Hungary; ^2^Radiobiology, University of the Witwatersrand, Johannesburg, South Africa; ^3^Institute of Human Physiology and Clinical Experimental Research, Semmelweis University, Budapest, Hungary

**Keywords:** modulated electro-hyperthermia, oncothermia, hyperthermia, selective heating, apoptosis, abscopal effect

## Abstract

**Background:** Modulated electro-hyperthermia (mEHT) is a variation of the conventional hyperthermia which selectively targets the malignant cell membranes in order to heat the malignant tissue and sensitize the tissue to oncology treatments. Although widely applied, the formulation of guidelines for the use thereof is still in progress for many tumors.

**Aim:** In this paper we review the literature on the effects of mEHT in cancer patients on local disease control and survival.

**Methodology:** Our review on data presents the collected experience with capacitive hyperthermia treatments with the EHY-2000+ device (OncoTherm Ltd., Germany). A literature search was conducted in Pubmed and articles were grouped and discussed according to: trial type, animal studies, *in vitro* studies, and reviews. Search results from Conference Abstracts; Trial Registries; Thesis and Dissertations and the Oncothermia Journal were included in the discussions.

**Results:** Modulated electro-hyperthermia is a safe form of hyperthermia which has shown to effectively sensitizes deep tumors, regardless of the thickness of the adipose layers. The technology has demonstrated equal benefits compared to other forms of hyperthermia for a variety of tumors. Given the effective heating ability to moderate temperatures, the improved tumor perfusion, and ability to increase drug absorption, mEHT is a safe and effective heating technology which can be easily applied to sensitize tumors which have demonstrated benefits with the addition of hyperthermia. Modulated electro-hyperthermia also appears to improve local control and survival rates and appears to induce an abscopal (systemic) response to ionizing radiation.

**Conclusion:** Based on clinical studies, the method mEHT is a feasible hyperthermia technology for oncological applications. Concomitant utilization of mEHT is supported by the preclinical and clinical data.

## Introduction

Moderate hyperthermia in oncology refers to the process of heating a tumor to within a range of 39–42.5°C in order to sensitize the tumor to oncology treatments (1). Although hyperthermia has been investigated in oncology since the early 1900s (2) there are still gaps in the knowledge and application of hyperthermia in many settings and in the effects of hyperthermia.

One example is the role of temperature in the treatment planning. Although an increase in temperature to above 43°C has direct tumor killing effects (3, 4), there are potential risks associated with high temperatures, such as enhanced blood flow to the surrounding tissues which may potentially aid dissemination of the malignant cells (5–7), and restricted blood flow within the tumor (8), reducing drug delivery to the tumor. Furthermore the homogenous heating of a tumor to a specified temperature is challenging due to the highly inhomogeneous nature of the tumor resulting in a variation in temperatures within the tumor from 37°C to in excess of 43°C, depending on the presence and size of necrotic areas within the tumor (3).

Despite the unanswered questions, local hyperthermia has shown to significantly improve local disease control in a variety of tumors (9), and offers a valuable addition to the basket of treatments available to treat localized disease. Unfortunately the survival benefit is not always as significant as the local disease control with the addition of hyperthermia (10–14) and a reduction in metastatic (systemic) disease is also needed in order to improve survival rates. This could be achieved with the induction of a systemic response to the treatment. Datta et al. (9) discusses the immunomodulating effects of hyperthermia and the potential for hyperthermia to promote an abscopal effect when combined with ionizing radiation (9).

**Figure 1:**
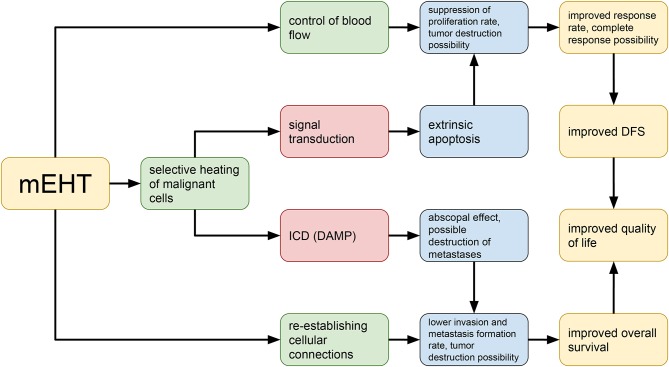
Basic principles of the pathobiological processes in the context of modulated electro-hyperthermia.

Modulated electro-hyperthermia (mEHT; trade name: oncothermia) is a relatively new method of hyperthermia proposed by Szasz et al. (15) which differs from conventional heating methods in that it focusses on the selective heating of the extracellular matrix and cell membranes in the malignant tissue (16, 17) rather than on the homogenous heating goal of conventional heating techniques (18). Technically, mEHT is a precious impedance matched the capacitive coupled device, its effects are summarized in Figure 1. This paper reviews the clinical literature on mEHT. To our knowledge this is the first paper to conduct a review of literature published in Pubmed, focussing on clinical publications with limited focus on “gray” literature.

## Methodology

A literature search was conducted in Pubmed using the string: “Cancer OR neoplasm OR tumor OR malignancies AND Oncothermia OR Oncotherm OR modulated electro-hyperthermia OR modulated electrohyperthermia” with truncated words included in the search. Articles were then classified as: Phase III randomized controlled trial (RCT); Non-randomized controlled trial; Phase I/II Trial; Animal studies; *in vitro* studies; “Gray” literature. The results were checked against a search for the same terms in the International Journal of Hyperthermia to make sure that all articles from the Journal were included in the search. “Gray” literature was included from Pubmed and from sources outside of the traditional commercial or academic channels and was further classified as: Expert reviews (from Pubmed); Case studies (from Pubmed); Conference Abstracts; Trial Registries; Thesis and Dissertations.

## Results

The Pubmed search returned 46 articles, of which five were excluded as they did not involve the Oncotherm methods. Of the 42 eligible articles, six were from the International Journal of Hyperthermia and all articles on the Oncotherm method in International Journal of Hyperthermia appeared in the Pubmed search. Table 1 summarizes and categorizes the literature reviewed.

**Table 1:** Summary of papers returned from the search string.

Deleted—not relevant (from Pubmed)	*n =* 5
Phase III randomized controlled trial (RCT)	*n =* 2
Non-randomized controlled trial (from Pubmed)	*n =* 1
Phase I/II Trial (from Pubmed)	*n =* 10
Animal studies and *in vitro* studies (from Pubmed)	*n =* 18
“Gray” literature	
Expert reviews (from Pubmed)	*n =* 7
Case studies (from Pubmed)	*n =* 3

A search in ClinicalTrials.gov returned four trials registered and currently underway investigating the Oncotherm method

1. Multicenter RCT of the Clinical Effectiveness of Oncothermia With Chemotherapy (Folfirinox or Gemcitabine) in Metastatic Pancreatic Cancer Patients (Seoul National University Bundang Hospital, Republic of Korea); ID: NCT02862015.
2. A Trial of Weekly Paclitaxel with Oncothermia and Weekly cisplatin With Oncothermia in Patients With Recurrent or Persistent Ovarian Cancer (Seoul National University Bundang Hospital, Republic of Korea); ID: NCT02344095.
3. Effect of Oncothermia on Improvement of Quality of Life in Unresectable Pancreatic Cancer Patients (Seoul National University Bundang Hospital, Republic of Korea); ID: NCT02150135.
4. Modulated Electro-Hyperthermia Plus Chemo-radiation for Locally Advanced Cervical Cancer Patients in South Africa (Charlotte Maxeke Johannesburg Academic Hospital, South Africa); ID: NCT03332069.

There is a substantial amount of information available in the form of conference papers, books and expert reviews. Seven reviews on mEHT are available in Pubmed, primarily addressing the theory, biophysics and preclinical work on mEHT and containing references to conference papers, books and other references not addressed in this paper. As this information is discussed in detail in other papers, it is not addressed in this report.

Hegyi et al. (19) provides a detailed description of the goals and benefits of mild heating, effects of hyperthermia and the theory of mEHT. Another paper by Hegyi et al. (19) describes the cellular effects of hyperthermia compared to Oncothermia. The authors propose that the lack of acceptance of hyperthermia was in part due to the controversial results seen in hyperthermia trials published at the time and conclude that mEHT can solve many of these challenges, such as decreased risks of dissemination, deep heating, and selective heating. At the time however, mEHT lacked the clinical data and data on long term outcomes needed to optimize the protocols and implementation of the treatment (19).

Szasz (20) reviewed the use of mEHT for lung cancer patients and found mEHT to be a safe treatment with the potential to increase survival benefits and quality of life (20). In a very detailed and thorough review, Roussakow (21) reported on the use of mEHT for the management of recurrent glioblastomas. The paper evaluated the economic effect of the use of mEHT in these patients and concluded that mEHT significantly improves survival of patients treated with dose-dense temozolomide (21/28 days regimen) while also demonstrating cost-effectiveness (21). Based on this review and publications by Wismeth et al. and Fiorentini et al. (22–24). Prieto and Linares propose further research on thermosensitive liposomes combined with mEHT to manage brain tumors (25).

The biophysics of mEHT is dealt with in detail by Fiorentini and Szasz (26). Modulated electro-hyperthermia combines the effects of electric fields and heating to damage the malignant tissue. The selection is based on the differences in the electrical properties between healthy and malignant tissue (26). According to conventional hyperthermia the dose is measured by the temperature achieved in 90% of the tumor. This requires monitoring by either intratumoural thermometers or MR-technology (18). However the dose of mEHT is not measured by the temperature achieved in the tumor, but rather by the energy deposited in the tumor required to induce the sensitizing and cell-killing effects (26). The mathematical models and dosing concepts are dealt with elsewhere in the literature (27–29). Andocs et al. (30) effectively summarized the evolution of mEHT from the preclinical setting to the clinical setting showing the advantages of the mEHT compared to classical heat treatments at the same temperatures (30).

### Safety of mEHT

The safety of mEHT has been demonstrated as a monotherapy in women with relapsed or refractory ovarian cancer. The aim of the study was to confirm the maximum tolerated dose of mEHT. Nineteen participants were treated with mEHT twice a week for 3 weeks, starting at 110 W and increasing the power to 130 W. No dose-limiting toxicities were noted with mEHT administered to the peritoneal region (31). An Italian study has also demonstrated the safety and tolerability of mEHT applied to a variety of solid tumors (32).

The effects of mEHT on the pharmacokinetics of nefopam (33) and fentanyl (34) were investigated in two randomized cross-over studies. The results showed that mEHT administered to the abdomen increased the absorption of nefopam administered orally and this resulted in an increase in the blood concentration of nefopam (33). This suggests that mEHT treatments may be used to increase the absorption of chemotherapy drugs at the heated site. The increase in overall exposure to oral transmucosal fentanyl citrate administered with mEHT was not associated with any clinical implications and is therefore considered safe to be administered in combination with mEHT (34).

Tumors in the brain and central nervous system are difficult to heat without risking damage to the healthy tissue. Two phase I/II studies have been conducted on mEHT applied to brain tumors. Fiorentini et al. (22) demonstrated the safety of mEHT in eight glioblastoma multiforme patients, two anaplastic astrocytoma (grade III) patients and two anaplastic oligodendroglioma. All patients had been previously treated with temozolamide-based chemotherapy and radiotherapy. Modulated electro-hyperthermia was applied up to 150 W without dose-limiting toxicities. The authors reported one complete remission and two partial remissions, a median duration of response of 10 months (range 4–32) and a 1 year survival rate 25%.

Wismeth et al. (24) confirmed the safety of mEHT with chemotherapy in a prospective single-arm Phase I trial involving 15 participants with high grade gliomas treated two to five times a week with mEHT (dose escalation protocol) combined with alkylating chemotherapy (ACNU, nimustin; 90 mg/m2). Participants were treated until dose-limiting toxicities developed or disease progression developed. Toxicities associated with mEHT included local pain, increased focal neurological signs, and increased intracranial pressure requiring mannitol or corticosteroids to resolve symptoms, however no dose-limiting toxicities developed (24).

Gadaleta-Caldarola et al. (35) reported excellent safety results following the use of mEHT combined with sorafenib for hepatocellular carcinoma. The authors investigated the combination as sorafenib inhibits cellular proliferation and angiogenesis and hyperthermia inhibits angiogenesis by damaging endothelial cells and increasing the expression of PAI-1 in the endothelial cells (36). The induction of apoptosis by mEHT has also been established in preclinical work (37–39). Twenty one participants with advanced hepatocellular carcinoma were enrolled and treated three times a week with mEHT for 6 weeks combined with sorafenib 800 mg on alternating days, followed by a 2 week break. The results showed stable disease in 50% of participants, partial response in 5 and 45% with progressive disease. Treatment related toxicities were associated with sorafenib and not directly attributed to mEHT and the addition of mEHT was well-tolerated and did not increase the sorafenib-related toxicities (35).

The intravenous administration of high doses of ascorbic acid is popular in the complementary and alternative fields of medicine. Ou et al. (40) showed that mEHT is safe to combine with intravenous ascorbic acid in non-small cell lung cancer patients in China. The researchers found that peak concentration of ascorbic acid was increased in participants treated with mEHT concurrently with the administration of ascorbic acid. However no dose-limiting toxicities were noted (40).

### Cervical Cancer

Hyperthermia has been investigated extensively for the treatment of cervical cancer patients (41). In patients who are unable to receive the prescribed dose of chemotherapy, hyperthermia can be combined with radiation (42) with similar outcomes to chemoradiotherapy (43). Two randomized controlled trials investigating mEHT appeared in the Pubmed search, both on cervical cancer.

Lee et al. (44) investigated the use of mEHT combined with platinum based chemotherapies compared to chemotherapy alone for locally recurrent or residual cervical cancer post-radiotherapy. The combined results from two prior Phase II trials investigating hyperthermia and platinum based chemotherapy for locally recurrent cervical cancer showed an overall response rate of 54% (45, 46). Franckena et al. (46) subsequently recommended that this combined treatment be applied as a standard treatment approach for cervical cancer patients with locally recurrent or residual disease within a previously irradiated area. In the study by Lee et al. (44), patients were randomized to receive chemotherapy (paclitaxel + cisplatin; paclitaxel + carboplatin; cisplatom + 5-fluorouracil; or cisplatin) with (*n* = 20) or without (*n* = 18) mEHT for local recurrent cervical cancer patients who had been previously irradiated. A total of 36 mEHT treatments were administered (three times per week) beginning at chemotherapy initiation. The overall response to treatment was significantly greater in the group of patients treated with mEHT + chemotherapy (*p* = 0.0461), and the difference remained significant at the evaluation conducted at the last follow-up visit (*p* = 0.0218) with a complete response of 15% in the chemotherapy group vs. 50% in the combined group (47).

Minnaar et al. (48) report on early results from their phase III RCT on the use of mEHT combined with chemoradiotherapy (CRT) for locally advanced cervical cancer (LACC) patients in South Africa. The study was the first to include HIV-positive patients in a hyperthermia trial and it was conducted in a resource-constrained setting. Participants were randomized to receive chemoradiotherapy (CRT) (cisplatin, external beam radiotherapy, and high dose rate brachytherapy) or CRT + mEHT (administered twice per week for an hour, immediately before external beam radiotherapy). At 6 months post-treatment, 101 participants were available for evaluation in each group. Six month local disease-free survival and 6 month local disease control were significantly higher in the mEHT Group (*n* = 39 [38.6%] and *n* = 40 [45.5%], respectively), than in the Control Group (*n* = 20 [19.8%]); *p* = 0.003 and *n* = 20 [24.1%]); *p* = 0.003). The authors reported that mEHT did not have any effect on the frequency of CRT-related early toxicities and the outcomes were not associated with the body mass index of the patients, suggesting that therapeutic effects were seen even in participants with thicker layers of adipose tissue in the treatment field. In a comparison between the pre-treatment 18F-FDG PET/CT scans of participants with extra-pelvic nodes to the post-treatment scans of the same participants, the percentage of participants who showed a systemic response to treatment, with resolution of all metabolically active disease (including extra-pelvic nodes which were not in the treatment fields, pelvic nodes, and the primary tumor), was significantly higher in the mEHT Group: 24.1% (*n* = 13), vs. the Control Group: 5.6% (*n* = 3; *p* = 0.007) regardless of HIV status (48). These results show mEHT is an effective hyperthermia technique producing results comparable to conventional hyperthermia, without unexpected toxicity. Preliminary results reported on 2 year disease-free survival showed a significantly higher disease-free survival in the mEHT group (49).

Lee et al. (44) showed that mEHT effectively improves the blood flow (measured by a Doppler ultrasound) to cervical tumors heated with mEHT in 20 patients. In their study the mean peri-tumor temperature was 36.7 ± 0.2 °C before heating and increased to 38.5 ± 0.8 °C at the end of heating for 60 min. This increase of roughly 2 °C is in line with the studies on other heating techniques applied to cervical tumors (50) and provides evidence of the radiosensitising effects for mEHT based on improved blood flow and subsequent improved oxygen in the tumoral environment.

### Brain Tumors

In a non-randomized, multicenter, retrospective controlled trial, Fiorentini et al. (51) analyzed the effects of mEHT as a palliative option for the management of relapsed malignant glioblastoma (GBM) and astrocytoma (AST). One hundred and forty nine consecutive participants who had relapsed after surgery, adjuvant temozolomide-based chemotherapy, and radiotherapy, were enrolled (glioblastoma: *n* = 111; astrocytoma: *n* = 38). Participants were treated with mEHT (progressing from 40 to 150 W using a step-up heating protocol for 20 min progressing to 60 min) or best supportive care, as indicated. Twenty eight (25%) glioblastoma participants and 24 (63%) astrocytoma participants received mEHT three times a week for 8 weeks. Imaging studies were conducted every 3 months to assess response rates. At 3 months, an objective response (complete and partial response) in the AST participants treated with mEHT was significantly higher than in the AST participants treated with best supportive care (45 vs. 6%; *p* < 0.005). Progressive disease was observed in 4 (18%) of the AST participants in the mEHT group compared to 9 (56%) participants in the best supportive care group. Glioblastoma participants in the mEHT group had a significantly higher overall positive response (complete and partial response and stable disease) at three months than the participants in the best supportive care group (54 vs. 19%; *p* < 0.05). The 5 year overall survival of AST participants in the mEHT group was 83%, compared to 25% in the best supportive care group and the GBM patients treated with mEHT had a 3.5% 5 year survival compared to a 1.2% 5 year survival in the best supportive care group. These results provide strong motivation for further investigations into the inclusion of mEHT in the palliative management of GBM and AST patients with relapsed disease (23).

### Peritoneal Metastases

Chinese hospitals frequently apply traditional Chinese medicine (TCM) combined with conventional treatments for a variety of disorders, including cancer. In a randomized Phase II trial in China (ClinicalTrials.gov ID: NCT02638051), mEHT combined only with TCM (*n* = 130) was compared to intraperitoneal chemo-infusion (IPCP) with cisplatin and fluorouracil (*n* = 130) administered twice, for patients with peritoneal metastases and malignant ascites. The “Shi Pi” herbal decoction was used for TCM and mEHT was applied every second day for 4 weeks. The overall response rate was 77.69% (101/130) vs. 63.85% (73/130) in patients treated with mEHT and IPCP, respectively, (*p* < 0.05) while the mEHT group reported less toxicity.

### Case Reports

Yeo (52) describes a case of a 75-year-old patient with stage IIIB non-small-cell lung cancer treated with radiotherapy and mEHT. The patient was not eligible for radical treatment and chemotherapy due to the age and performance status of the patient. Radiotherapy was administered in 36 fractions (total 64.8Gy) and mEHT was administered twice per week immediately before radiation (total 12 sessions). No dose-limiting toxicities developed and mEHT was well-tolerated. The follow-up imaging studies showed complete tumor response and the patient was disease free at 18 months post-treatment (52). The concurrent administration of bevacizumab and mEHT in a patient with bone metastasis from non-small cell lung carcinoma resulted in disease stabilization and improved pain management (53). Lee et al. (54) reported on a patient treated with mEHT, thymosin-α1, and a herbal treatment for lung metastases from a Wilms tumor in a patient previously treated with radiotherapy and chemotherapy. Disease stabilization of the lung metastases was noted on post-treatment CT scans (54).

**Figure 2:**
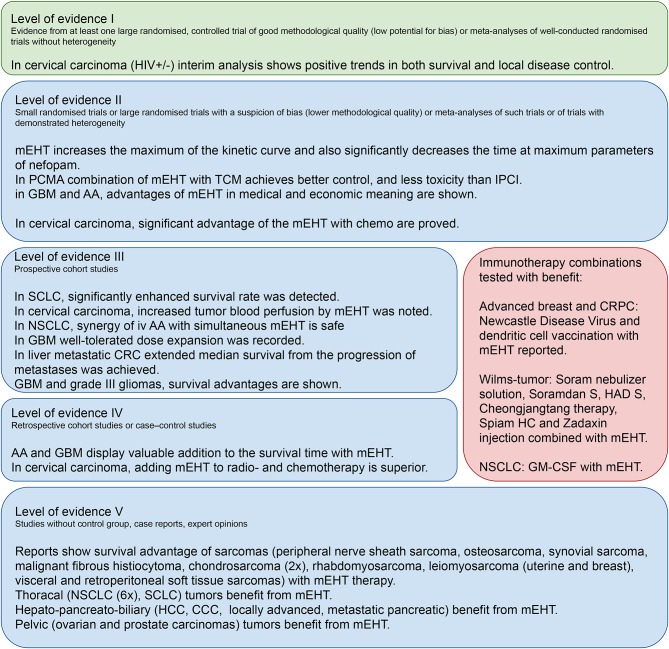
The verification and clinical evidence behind the modulated electro-hyperthermia clinical studies.

The above research facts are verified in the clinical applications showing various clinical results (Figure 2).

## Conclusion

One of major challenges in hyperthermia is the safe treatment of tumors of the central nervous system and brain. Modulated electro-hyperthermia has shown safety in brain tumors (24) and efficacy for relapsed brain tumors as a monotherapy for palliative management (51). Capacitive heating is cited as being unable to effectively heat deep tumors (55, 56) however mEHT has demonstrated safety and improved outcomes after the treatment of deep seated pelvic (cervical) tumors, even in obese patients (48). No dose limiting toxicities were noted in Phase I/II studies on peritoneal metastases in patients with recurrent disease from ovarian cancer, lung treatments (40) and treatments to the liver (35).

Modulated electro-hyperthermia is a safe form of hyperthermia which has demonstrated equal benefits compared to other forms of hyperthermia for a variety of tumors, including deep pelvic tumors. Minnaar et al. (48) showed that the body mass index of participants in their study was not associated with treatment outcomes, suggesting that mEHT effectively sensitizes deep tumors, regardless of the thickness of the adipose layers. Modulated electro-hyperthermia also appears to induce an abscopal (systemic) response to ionizing radiation (48) which is in line with the immunomodulating effects of mEHT described in the preclinical studies (9, 57). There does not appear to be an increased risk in disease dissemination and early results indicate improved disease free survival in patients treated with mEHT (23, 47, 48).

Given the ability of mEHT to heat tumors to within the moderate hyperthermia heating range [as demonstrated in a porcine model (58), other animal models (59–61). and human (44) studies], the improved perfusion, and ability to increase drug absorption, mEHT is a safe, and effective heating technology which can be easily applied to treat tumors which have demonstrated benefits with the addition of hyperthermia.

## Author Contributions

AS and GPS: conception and design. AS, CM, GS, GPS, and MD: writing the manuscript. AS: construction of images.

### Conflict of Interest

The authors declare that the research was conducted in the absence of any commercial or financial relationships that could be construed as a potential conflict of interest.
